# Classification of Fatigued and Drunk Driving Based on Decision Tree Methods: A Simulator Study

**DOI:** 10.3390/ijerph16111935

**Published:** 2019-05-31

**Authors:** Ying Yao, Xiaohua Zhao, Hongji Du, Yunlong Zhang, Guohui Zhang, Jian Rong

**Affiliations:** 1 Beijing Key Laboratory of Traffic Engineering, College of Metropolitan Transportation, Beijing University of Technology, 100 Pingleyuan, Chaoyang District, Beijing 100124, China; yaoying@emails.bjut.edu.cn (Y.Y.); jrong@bjut.edu.cn (J.R.); 2 Autonomous Driving unit, Baidu.com, Inc, No. 10 Xibeiwang East Road, Haidian District, Beijing 100193, China; hongji_du@hotmail.com; 3 Zachry Department of Civil Engineering, Texas A&M University, 3136 TAMU, College Station, TX 77843, USA; yzhang@civil.tamu.edu; 4 Department of Civil and Environmental Engineering, University of Hawaii at Manoa, 2540 Dole Street, Holmes 338, Honolulu, HI 96822, USA; guohui@hawaii.edu

**Keywords:** fatigued driving, drunk driving, driving performance, roadway geometry, decision tree

## Abstract

It is a commonly known fact that both alcohol and fatigue impair driving performance. Therefore, the identification of fatigue and drinking status is very important. In this study, each of the 22 participants finished five driving tests in total. The control condition, serving as the benchmark in the five driving tests, refers to alert driving. The other four test conditions include driving with three blood alcohol content (BAC) levels (0.02%, 0.05%, and 0.08%) and driving in a fatigued state. The driving scenario included straight and curved roads. The straight roads connected the curved ones with radii of 200 m, 500 m, and 800 m with two turning directions (left and right). Driving performance indicators such as the average and standard deviation of longitudinal speed and lane position were selected to identify drunk driving and fatigued driving. In the process of identification, road geometry (straight segments, radius, and direction of curves) was also taken into account. Alert vs. abnormal and fatigued vs. drunk driving with various BAC levels were analyzed separately using the Classification and Regression Tree (CART) model, and the significance of the variables on the binary response variable was determined. The results showed that the decision tree could be used to distinguish normal driving from abnormal driving, fatigued driving, and drunk driving based on the indexes of vehicle speed and lane position at curves with different radii. The overall accuracy of classification of “alert” and “abnormal” driving was 90.9%, and that of “fatigued” and “drunk” driving was 94.4%. The accuracy was relatively low in identifying different BAC degrees. This experiment is designed to provide a reference for detecting dangerous driving states.

## 1. Introduction

Fatigue and alcohol have been identified as major factors in road accidents in many countries [1,2,3]. Driver fatigue is involved in 10–15% of all severe crashes according to a conservative estimate [4]. Alcohol consumption results in an annual death of 2.5 million people either from alcohol-related diseases or from accidents related to alcohol-impaired behavior [5].

A lot of studies investigated the effects of fatigue and alcohol on driver’s performance. Some of these work well in showing how fatigue influences driving performance measures such as speed, lane position, and steering wheel movements [6,7,8]. Fillmore et al. [9] claimed that alcohol significantly impaired driving performance with respect to deviation of lane position, line crossings, steering rate, and driving speed. As to the comparison between fatigued driving and drunk driving, Arnedt et al. [10] demonstrated that 18.5 and 21 hours of wakefulness produced decrements in driving performance measures similar to blood alcohol contents (BACs) of 0.05 and 0.08%, respectively. Alcohol produced changes in mean tracking and tracking variability that was similar to those produced by comparable periods of prolonged wakefulness. Speed variability was also affected by alcohol and by prolonged wakefulness.

Meanwhile, the characteristics of road geometry influence driving performance by affecting arousal, alertness, and information processing [11,12,13]. Driving speed, lane position, and accident rate can also be affected by road geometry [14]. Regarding the influence of road conditions on driving performance, it is reasonable to assume that different road conditions may affect the influence of fatigue and alcohol on driving performance. A few studies have pointed out the different characteristics of fatigued driving and drunk driving under different road geometries. Desmond and Matthews [15] found that when the task was relatively difficult (curved road), fatigued drivers were able to cope with increased demands. However, when the task was easy (straight road), the performance tended to deteriorate, implying that fatigued drivers were failing to mobilize their effort effectively. Drivers are aware of a general decrement in their performance due to alcohol or fatigue, and their way to handle the decrement is relative to the demands of the road.

Dawson and Reid [16] suggested that sleepiness and alcohol each produced performance decrements, and thus a common metric could be established between the impairments. Using a tracking task, they found that impairments in psychomotor performance after 24-h sustained wakefulness were of the same magnitude as those at BAC 0.10%. Moderate levels of prolonged wakefulness can produce decrements in performance of a magnitude similar to BACs prohibited by law. Previous researchers have examined the consistency of the performance impairment produced by fatigue and by alcohol. However, they did not clarify how to distinguish fatigued driving from drunk driving with different BAC levels, taking driving performance as indicators. Therefore, we explored the effects of drinking at different BACs and fatigue on driving performance under different roadway geometries in the previous paper [17]. Now, we will discuss how to distinguish fatigued driving and drunk driving in the present study. 

In relevant studies, researchers have acquired information, including road environment and vehicle state, using sensor equipment. They also applied a machine learning algorithm to distinguish driving performance before making a decision [18]. In the early 1990s, Hernandezgress [19] integrated multisensory information and applied principal component analysis and neural network to identify if the driving performance was normal. In 2005, Tsironis et al. [20] used decision trees to build a detective model for aberrant driving characteristics, and assessed the accuracy of results using randomized testing. Later, with the dynamic Bayesian network and the junction tree algorithm, Kumagai [21] improved the accuracy of driving behavior prediction.

A decision tree is a structure that can be used to divide up a large collection of records into successively smaller sets of records by applying a sequence of simple decision if-then rules [22]. Decision trees merge both data exploration and modeling, so they are useful for exploring data to gain insight into the relationships of a large number of input variables to a target variable. According to Breiman et al. [23], a popular approach to tree-structured modeling consists of two separate phases: growing and pruning. In tree growing, data are split into small nodes, referred as “child nodes”. Because the ultimate goal of tree growing is a wholesome outcome, tree growing is limited by sample size, node homogeneity, or stopping rules. After the trees have grown, algorithms are used to prune the tree back. Tree pruning is usually guided by cross-validation within the training data set [24].

Analyzing the influences of fatigue and alcohol consumption on driving performance in different road alignments could help to distinguish the characteristics of fatigued driving and drunk driving. The objectives of this study are to how to classify and predict fatigued driving and drunk driving under different road geometries through driving performance indicators on the basis of the decision tree. A decision tree can graphically depict the relationship between risky driving behavior and the influencing factors. More importantly, decision trees could avoid the inherent problems occurring in the multivariate regression models and provide high prediction accuracy. Therefore, this study employs decision tree to analyze the relationship between risky driving status and driving performance characteristics under different roadway geometries. The results are expected to verify a suitable method for distinguishing fatigued driving and driving at different alcohol consumption levels under different roadway geometries. We expect that our findings can provide accurate benchmarks to monitor drivers’ behavior and performance, so as to prevent injuries and reduce causalities caused by fatigued driving and drunk driving.

The remainder of this paper is organized as follows: literature review first, followed by materials and methods, results, discussion, conclusions, and future research directions.

## 2. Literature Review

### 2.1. Fatigue Driving

The research on fatigued driving detection includes the following methods: fatigue detection based on subjective evaluation, physiological parameters, drivers’ facial features, and vehicle operating status.

In terms of fatigued driving detection based on subjective evaluation, the evaluation methods mainly include self-evaluation and others’ evaluation. Among them, self-evaluation refers to filling out some questionnaires about the fatigue degree, such as the Karolinska Sleepiness Scale (KSS), the Stanford Sleepiness Scale (SSS), etc. Another evaluation showed that some professionals evaluated the degree of fatigue of drivers according to their facial and head posture characteristics [25]. 

Fatigue detection based on physiological parameters has also been extensively studied. Jap et al. [26] assessed the four types of electroencephalography (EEG) activity: delta (δ), theta (θ), alpha (α), and beta (β), to detect fatigue. Patel et al. [27] presented an artificial intelligence system which could detect the early onset of fatigue in drivers using heart rate variability (HRV) as the human physiological measure. Katsis et al. [28] proved the metrics of surface electromyograms such as mean frequency (MNF), median frequency (MDF), and the signal of root mean square (RMS) amplitude are reliable fatigue indicators to detect fatigue driving.

At the same time, there are some methods to detect fatigue driving through drivers’ facial features. Ji et al. [29] used remotely located charge-coupled-device cameras equipped with active infrared illuminators to acquire video images of the driver. The fatigue state of drivers can be judged by extracting their visual features in real time. Eriksson et al. described how to find and track the eyes, and they proposed a method to judge whether the eyes are open or closed so as to identify the fatigue state of drivers.

When drivers are driving with fatigue, abnormal fluctuations will appear in the control of the vehicle. Therefore, there are also some studies to identify fatigued driving by detecting the vehicle operating status. Friedrichs et al. [30] extracted 11 indexes of the steering wheel angle and vehicle lateral position, and a fatigued driving detection model was established based on a neural network which achieved high detection accuracy. Krajewski et al. [31] used advanced signal processing procedures for feature extraction of fatigued driving. Five machine learning methods (e.g., Support Vector Machine, K-Nearest Neighbor) were used to classify slight fatigue and strong fatigue.

### 2.2. Drunk Driving

The most common identification method for drunk driving is to detect the driver’s blood alcohol concentration (BAC) and breath-alcohol concentration [32,33]. At the same time, there are also some other methods to detect drunk driving, such as monitoring driver behaviors (e.g head and eye or pupil movements), and monitoring vehicle dynamics and behavior.

In terms of monitoring driver behaviors (e.g head and eye or pupil movements) to detect drunk driving, Liu et al. [34] investigated the effects of different breath alcohol concentrations upon driver driving behavior and subsidiary task performance. Hammoud et al. [35] proposed a vision detection algorithm by locating the eye position, tracks its position and movement over time. By applying this method, the drinking state of drivers could be detected.

Drunk driving detection methods based on vehicle dynamics and behavior have also been extensively studied. Zhao et al. [36] extracted seven significant indicators of driving performance, and drunk driving was identified by the Fisher Discriminant Method. Meanwhile, Zhao et al. [37] analyzed the driving behavior data under different BAC levels; the results showed that compared with normal driving, the average speed, standard deviation of the speed, and standard deviation of the lane line position are significantly changed when drunk driving. Dai et al. [38] extracted typical drunk driving patterns by using the data collected from the sensors of a mobile phone. Once any evidence of drunk driving is present, the mobile phone will automatically alert the driver or call the police for help well before an accident actually happens. 

Because drunk driving is illegal and there is a high risk of real car experiments, driving simulators are often used for drunk driving experiments.

### 2.3. Driving Simulator Study

Experiments based on driving simulator driving represent a safe, convenient, and controllable way to evaluate driving behavior. There have been many studies on driving behavior evaluation based on driving simulators. Bella et al. [39] conducted a driving simulator study comparing driver speed behavior during daytime and nighttime driving and model operating speeds and speed differentials, identifying significant factors that influence speed behavior under different lighting conditions. Casutt et al. [40] used driving simulator to analyze the driving behavior characteristics of older drivers and developed a screening system to identify unsafe older drivers. Tuokko et al. [41] analyzed characteristics of driving behavior among people of different ages, genders, and psychosocial and health characteristics. Jurecki [42] provided an overview of the research into the driver behavior in simulated near collision situations and explored the relationship between the intensity of the driver response (braking, steering), the driver response time, and the time-to-collision. Dulebenets et al. [43,44] presented a mixed-integer programming model that assigns individuals, including vulnerable population groups, to emergency shelters through evacuation routes during the available time periods. Man-Son-Hing et al. [45] assessed the risk of driving behavior in dementia patients by using the driving simulator. Wu et al. [46] used driving simulator to conduct eco-driving training for drivers, and the results showed that fuel consumption and emission were significantly reduced after training. Yao et al. [47] explored the relationship between the driver’s visual features and driving behaviors of distracted driving by using driving simulator, and developed a random forest method to classify distracted driving behaviors. Yan et al. [48] conducted a simulator validity study from two perspectives: a traffic parameter (speed) and a safety parameter (crash history). By comparing the speed data observed in field experiments and simulator experiments, it was found that there was consistency between them, which verifies the effectiveness of driving simulator in safety evaluations. 

The application of driving simulator lays a foundation for fatigue driving and drunk driving identification in this paper.

## 3. Materials and Methods 

### 3.1. Participants 

It has been found that at the same BAC levels, young adult drivers take a greater risks than older ones [49]. A total of 22 healthy young male objects were recruited to participate in the research. The average age was 25 (SD = 4.1, range = 20–35 years). Twelve of the 22 participants were professional drivers. All participants had possessed a valid driver license for no less than 3 years (average = 3.6), and they drove 4000–70,000 km/year (median = 8640). To explain briefly, the homogeneous sample of subjects selected intended to minimize any bias attributable to sample heterogeneity, as numerous studies have demonstrated that driving performance is mainly affected by age. This research contained different professions and ages so that the fatigued driving and drunk driving behavior of various type of populations, in general, can be reflected. The admission criteria include a regular circadian rhythm and no sleep disorders. All the drivers agreed and signed an informed consent before participating in the study, and they were paid RMB 1000 after completing the experiment.

### 3.2. Apparatus

#### 3.2.1. Equipment

**Figure 1:**
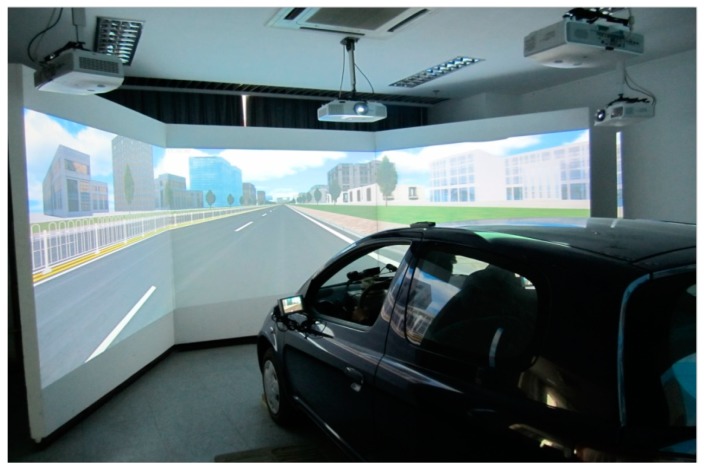
Driving simulator.

The experiment was conducted in an AutoSIM fixed-base driving simulator. In this driving simulator, the road scenario is projected onto three large screens in front, providing a 130-degree field of view, with two side mirrors and one rear view mirror showing a very realistic view (see Figure 1). This sampling period of simulator is 30 Hz. The simulator allows researchers to record the intensity of drivers’ actions on the brake, accelerator pedal, and steering wheel. Additionally, the simulator provides a number of other parameters that describe traveling conditions, such as the relative lateral position, the longitudinal speed, and acceleration. The sampling frequency of the driving simulator in this experiment is 30Hz. The validation and assessment for the simulator used in the study were conducted by Ding et al. [50]. Another apparatus was a blowing-type Breath Alcohol Concentration (BrAC) detector, and the detector was identical to that used by traffic police in Beijing. 

#### 3.2.2. Driving Scenario

**Figure 2:**
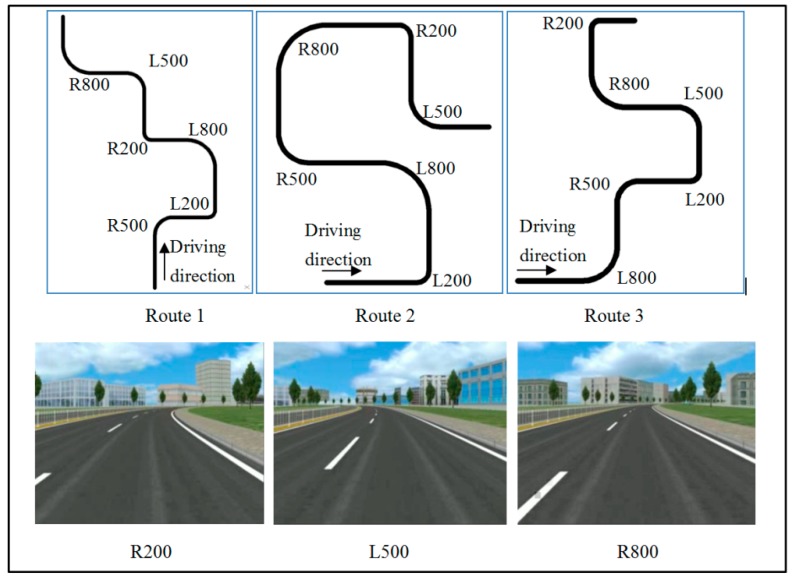
Three routes and simulated scenes.

Three various routes were designed for the experiment to avoid participants’ familiarity with a single route, shown in Figure 2 (L represents left and R represents right). The routes included curves with two turning directions (i.e., left and right), and three radii (i.e., 200, 500, and 800 m). Two adjacent curves were connected by a 2-km-long straight road. All roads in the scenarios were divided highways with two 3.75-m-wide lanes on both sides. 

### 3.3. Experimental Design and Procedure

To assess the effects of fatigued driving and different BACs on driving performance, participants were required to conduct experiments in five different states: alert, fatigued, and with BAC levels of 0.02%, 0.05%, and 0.08%. Each participant participated the experiments five times on five separate days. To avoid the residual effects of alcohol dose, the participants were required to participate experiments at three BAC levels of 0.02%, 0.05%, and 0.08% at intervals of 3, 5, and 7 days, respectively. All participants took the five driving tests in a random order to counterbalance the sequence effect.

The experiment time was designed according to the drivers’ sleep rhythm. The fatigued driving test was conducted at 02:00 h. None of them slept or took a nap during the daytime. They were asked to stay awake for about 18 hours before the fatigued driving test. The alert and drunk driving experiments were carried out between 14:00 h and 15:00 h. In addition, the participants were asked to have a good rest before the experiments to avoid the influence of fatigue. According to Watson’s [51] research, we calculated alcohol dose for each subject to achieve the expected BAC level. Participants were asked to fill out the seven-point Stanford Sleepiness Scale (SSS) questionnaire before and after each driving test. The SSS questionnaire [24] has seven degrees of sleepiness, including (1) feeling active, vital, alert, or wide awake; (2) functioning at high levels, but not at peak, able to concentrate; (3) awake, but relaxed, responsive but not fully alert; (4) somewhat foggy, let down; (5) foggy, loss of interest in remaining awake, slowed down; (6) sleepy, woozy, fighting sleep, desire to lie down; and (7) no longer fighting sleep, sleep onset soon, having dream-like thoughts. Caffeinated beverages, drugs, and alcohol were prohibited for 12 hours before each test. The detailed test procedure was described in our previous study [17,52].

### 3.4. Data Collection and Analysis

All fatigue and BAC levels of these 22 participants in every session were collected. For each session, researchers measured the BAC levels at the beginning and the end of the driving task. The mean of the two numbers was recognized as the BAC level during this simulated driving process. The fatigue degrees in each situation were presented by the SSS questionnaire results. The results showed that the drunk driving tests were not affected by fatigue, and the fatigued driving tests were conducted while the drivers were indeed fatigued.

**Figure 3:**
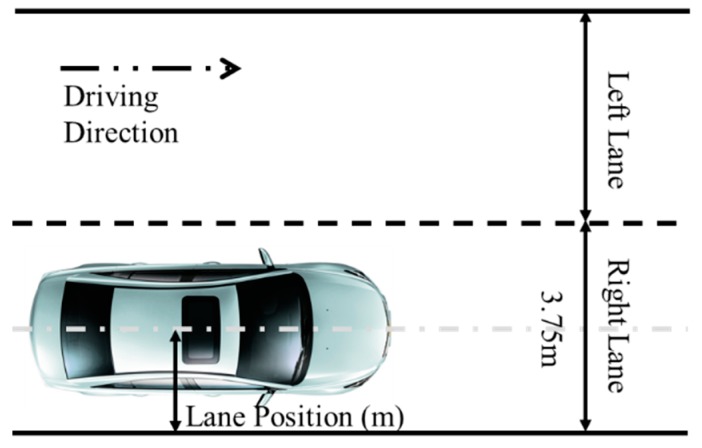
Description of vehicle lane position. Note: Lane position was defined according to the distance between the center of the vehicle and the edge line (0.2-m width) of the right lane: 1.9–2.2 m = driving in the middle of the right lane; 2.8 m and over = deviation from lane to the left lane; 1.3 m and under = deviation from lane to the right shoulder.

The driving simulator recorded continuously all the 22 participants’ data, including speed and lane position of five different states for every run. All data values were aggregated for the curves and straight roads, separately. A curve road segment is a road section from the entry point to exit point of the curve. A straight segment is a road section from 200 m after the exit point of the curve to 200 m before the next entry point of the curve. The mean and standard deviation of driving performance measures were calculated for each section, including the average speed (SP_AVG), the standard deviation of speed (SP_SD), the average of lane position (LP_AVG), and the standard deviation of lane position (LP_SD) (defined in Figure 3). These indexes (SP_AVG, SP_SD, LP_AVG, LP_SD) are obtained by calculating the mean or standard deviation of all sampling points of straight road segment and curve road segment. In general, the data were analyzed with repeated measures analyses of variance (ANOVA). In straight segments, researchers took the driving state as the only factor and conducted the ANOVA to verify the effects of different levels of BAC and fatigue on driving performance. As for curves, the repeated measures ANOVA was conducted for every driving performance measure, with drivers’ states (S: alert, BAC 0.02%, BAC 0.05%, BAC 0.08%, and fatigued), curve direction (D: right and left), and radius (R: 200 m, 500 m, and 800 m) as variables.

### 3.5. Introduction of Previous Research

The previous study showed that different BAC levels and fatigue influence drivers’ driving performance, including SP_AVG, SP_SD, LP_AVG, LP_SD [17]. Fatigued driving and drunk driving behavior also varied under different road geometries [17].

**Figure 4:**
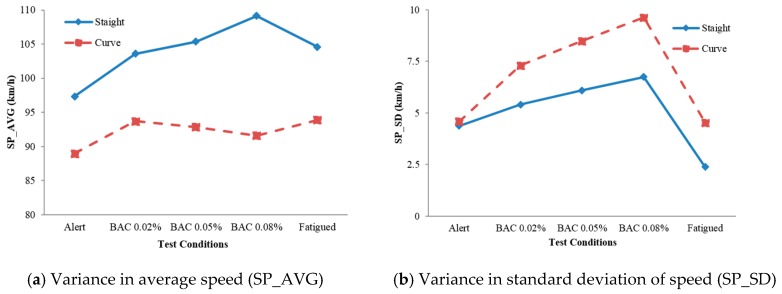

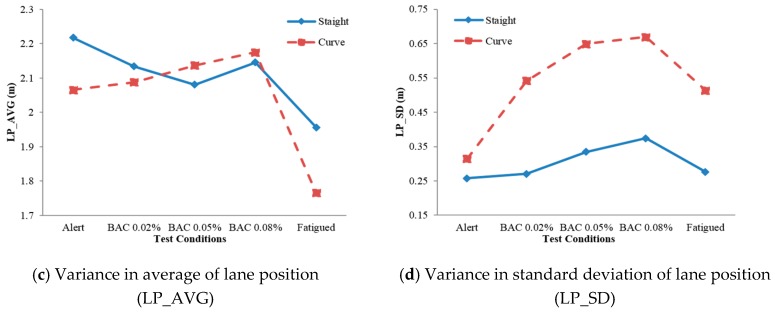
Variance in driving performance under straight and curve segments [17]. Note: (**a**) Variance in average speed (SP_AVG); (**b**) Variance in standard deviation of speed (SP_SD); (**c**) Variance in average of lane position (LP_AVG); (**d**) Variance in standard deviation of lane position (LP_SD).

As depicted in Figure 4, the trends of effects imposed by alcohol on SP_AVG and LP_AVG in curves (dotted lines) were different from that in straight segments (solid lines). In addition, the range of variance caused by alcohol and fatigue in SP_SD and LP_SD was more significant in curves than in straight segments. The following part intended to further find out the differences among various driving states in different curves.

Drivers’ states (S), radius (R), and turning direction (D) were extracted as factors to conduct repeated measures ANOVA. The results presented in Table 1 revealed that the main effects for drivers’ state, direction and radius were significant with respect to SP_AVG in curves. The significant main effect of the driver’s state on SP_AVG indicated that SP_AVG could be used to manifest the difference among different driving states. Significant interactions between drivers’ state and radius for SP_AVG were supposed to be caused by the combined effects of high alcohol consumption and sharp curves. The SP_SD was significantly affected by drivers’ state because the effects of fatigue on SP_SD were significantly different from the effects of alcohol consumption. The SP_SD became higher as the BAC level rose. The SP_SDs in drunk driving with BAC 0.05% and 0.08% were significantly higher than in alert driving. There was a significant difference in SP_SD between BAC 0.02% and BAC 0.08%. 

The results also indicated that LP_AVG was significantly affected by drivers’ state and turning direction. The LP_AVG was significantly lower in the fatigued state than that in other states (see dotted line in Figure 4c). This means that drivers would drive further toward the right of the lane under the influence of fatigue. There was a significant interaction between drivers’ state and turning direction on LP_AVG, which indicated that the effects of fatigue and alcohol consumption make drivers move closer towards the turning side. The results also showed that LP_SD was significantly affected by drivers’ state and radius. The LP_SD was significantly higher at BAC 0.08% than those in the alert state, BAC 0.02%, and fatigued state (Figure 4d).

In straight segments, higher SP_AVG could only distinguish drunk driving and fatigued driving from alert driving, yet it failed to separate drunk driving from fatigued driving. Nor could it distinguish drunk driving at different BAC levels. In curves, all driving performance measures were significantly affected by driver’s states. However, when the features of roadway geometry, such as radius and turning direction, had been ignored, it was impossible to differentiate one driving state from another accurately. There were five states (alert, BAC 0.02%, 0.05%, 0.08%, and fatigued), three radii, and two directions, and thus 30 (5 × 3 × 2) conditions in total. If we took one of the four driving performance measures in any two conditions and made a comparison, we could get $C_{30}^{2}=435$ results from the paired sample *t*-test. We, therefore, employ the driving performance measures described in the above sections as the variables of the CART which are used to classify driver’s risky behaviors. As described above, road geometries should be considered before distinguishing different driving states.

### 3.6. Decision Trees

We used decision trees (DTs) to further classify relationships among the independent and dependent variables. Classification and Regression Trees (CART) were proposed by Breiman [21]. CARTs are constructed for classifying the data, and the classification error decreases as the number of tree nodes increases (or the tree complexity increases). On the other hand, after dropping to a certain number of terminal nodes, cross validation results start increasing. To find the “optimal” tree size, the trade-off between the tree complexity and misclassification cost is measured by comparing tree costs with cross validation and resubstitution [53]. Because of its simplicity and high accuracy, the CART algorithm is employed to reproduce a decision tree using IBM SPSS Statistics 20.0 (IBM, Armonk, NY, USA) in this study. The CART algorithm constructs the decision tree by processing all input variables and expanding the tree to fit/classify the target variable. The CART procedure consists of three steps: first, growing a large tree structure; then pruning it to obtain a sequence of nested subtrees, and finally selecting the best tree model from the subtree sequence via a validation method. We chose driver status as the dependent variable and picked SP_AVG, SP_SD, LP_AVG, and LP_SD as independent variables for the decision tree model. The decision trees are developed based on the following rules:

- Each tree is composed of a maximum of five depth levels.
- There must be a minimum number of five cases in a child node and a minimum number of 10 cases for the parent node.
- Use “Gini” as the impurity measure.
- The minimum change in improvement is 0.0001.

Gini is a method of feature selection, which represents the possibility of a randomly selected sample being misclassified in a subset. The Gini coefficient is calculated as follows:

(1) $$Gini\left(D\right)=\sum_{i=1}^{n}p_{i}\left(1-p_{i}\right)=1-\sum_{i=1}^{n}p_{i}^{2}$$

where *D* represents all samples of the data set, and $p_{i}$ represents the probability of each category.

The number of sample folds was set to 10 when cross-validation was used to test the CART model.

## 4. Results and Discussion

### Decision Tree

The results of ANOVA above showed some characteristic effects of different BAC levels and fatigue on driving performance under different roadway geometries. These characteristic effects were fundamental for the detection of drinking and fatigued driving. Driving performance measures that vary significantly on the same road segment could be used together to distinguish driving states.

We employed the CART algorithm to classify, identify and predict fatigued driving and drunk driving. First, we assigned different values to label different test conditions: 0 = “alert”, 1 = “fatigued”, 2 = “BAC 0.02%”, 5 = “BAC 0.05%”, 8 = “BAC 0.08%”, 10 = “drinking” (including BAC 0.02%, 0.05% and 0.08%), and −1 = “abnormal”. The accuracy of predicted driving status by CART under different road geometries is shown in Table 2. Predicted percent correct is defined as follows:

(2) $$PPC_{i}=\frac{CS_{i}}{S_{i}}\times 100\%$$

where i represents driver status (alert, fatigued, BAC 0.02%, BAC 0.05%, and BAC 0.08%), and $CS_{i}$ represents the sample size in which the predicted results are consistent with the actual results, $S_{i}$ represents the total number of samples in the current driver status. 

The SP_AVG, SP_SD, LP_AVG, and LP_SD on each segment are used to identify the driver’s state. As shown in Table 2, prediction accuracy fluctuates violently under the same state on different road geometries. For instance, when the driver is alert, the prediction accuracy on the straight segment is only 27.3%, but the rate is more than 90% on L500 and R200 curves. A similar conclusion can also be achieved under different drivers’ states on the same road geometry. For example, on the L200 curve, the prediction accuracy of BAC 0.05% is zero, while the accuracy rate of BAC 0.02% is 90.9%. Despite the accuracy fluctuation, as seen in Table 3, the resubstitution risks of the CART model are all beyond 30% and the cross-validation risks are over 60%, indicating that accuracy and reliability of classification are quite low.

Driving is a continuous process on all types of road geometries, but, as proved in the data analysis section, driving performance differs on different segments. To find the proper road geometries and driving performance indicators that serve best to identify drivers’ states, we input all the following indicators on straight segments and all six curves as the dependent-variables of CART: S_SP_AVG, S_SP_SD, S_LP_AVG, S_LP_SD, L200_SP_AVG, L200_SP_SD, L200_LP_AVG, L200_LP_SD … R800_SP_AVG, R800_SP_SD, R800_LP_AVG, and R800_LP_SD, with 28 in total. The X column in Table 2 and Table 3 shows that the overall percentage is improved, but the identification accuracy of BAC levels is still quite low, and the resubstitution and cross-validation risks of predicted classification of CART model are not improved significantly. Thus, the research methodology and result cannot be applied in real life.

Since the decision tree is an efficient and accurate method to process binary data, we decided to divide all the five states into two parts in every process. Firstly, to reduce data dimension, fatigued driving and drunk driving at three BAC levels are recognized as the “abnormal state”. The “abnormal state” is the opposite of the “alert state”, and the two states are analyzed by a decision tree. Then, a new decision tree is established to classify the “abnormal state”, and the two independent states are “fatigued driving” and “drunk driving”. The third decision tree is established to classify “drunk driving”, and the independent variables are three different BAC levels.

**Figure 5:**
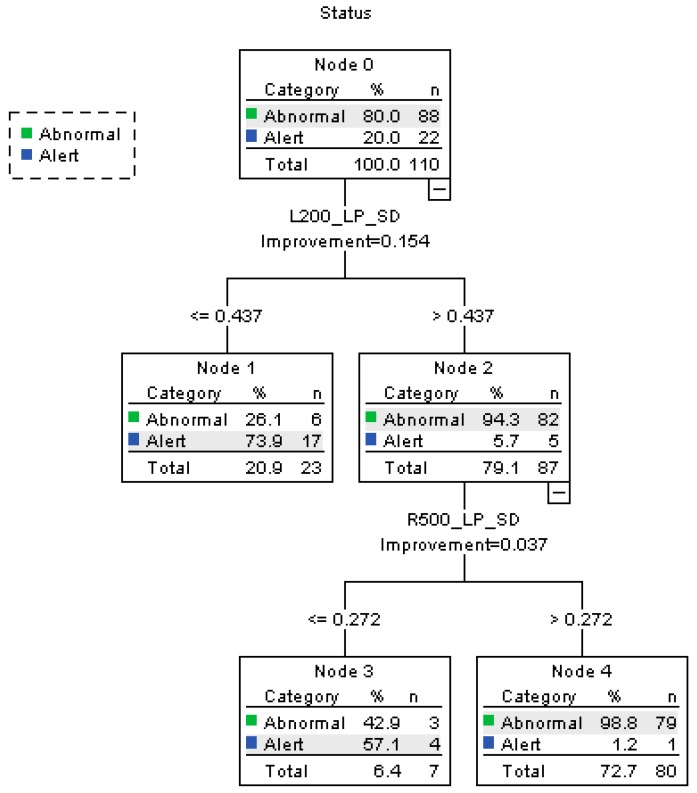
Decision tree diagram for the CART model of alert vs. abnormal.

Figure 5 displays the results of the first CART model of “alert” and “abnormal” statuses. Three terminal nodes were produced by the model. The tree’s characteristics are listed as follows:

(1) On the curve to the left with a radius of 200 m, when L200_LP_SD ≤ 0.437, 20.9% of the participants fall into “alert” category. The rest 79.1% are under the “abnormal” state.
(2) When L200_LP_SD is larger than 0.437, 94.3% of the participants under abnormal state are correctly classified.
(3) On a curve to the right with a radius of 500 m, when R500_LP_SD > 0.272, 72.7% of the participants are classified as “abnormal”. The percent correct of the abnormal state is 98.8%.
(4) As a conclusion, the accuracy rate of the alert state is 95.5% and abnormal state 89.8%. The overall accuracy rate is 90.9%, the precision is 98.75%, and the recall is 89.77%. The risk estimate of resubstitution is 0.091 and the risk estimate of cross-validation is 0.164.

The results show that LP_SD in different curves could be used to distinguish abnormal driving from normal driving with high accuracy.

**Figure 6:**
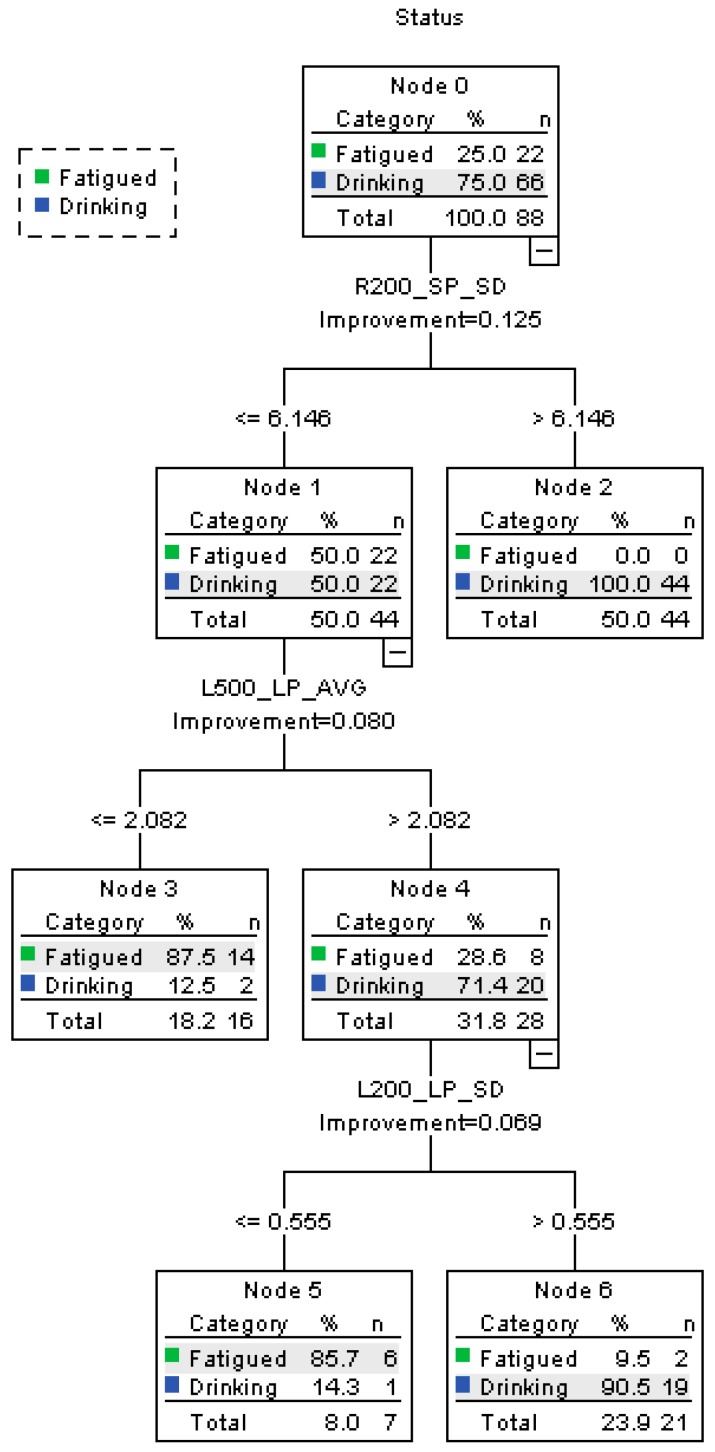
Decision tree diagram for the CART model of fatigued vs. drunk driving.

Figure 6 displays the results of the second CART model of fatigued and drinking statuses. Four terminal nodes were produced by the model. The tree’s characteristics are listed as follows:

(1) On a curve to the right with a radius of 200 m, when SP_SD > 6.146, 50% of the participants are classified as drunk driving, and none of them are fatigued.
(2) When R200_SP_SD ≤ 6.146, the other 50% of the participant also are considered as “drunk”. Then on a curve to the left with a radius of 500 m, where L500_LP_AVG ≤ 2.082, 18.2% of the drivers are classified as performing fatigued driving. The accuracy rate is 87.5%. When L500_LP_AVG > 2.082, 31.8% of the participants are judged as having drunk alcohol.
(3) When L200_LP_SD ≤ 0.555, 8% of the participants are reclassified as “fatigued”. When L200_LP_SD > 0.555, the other participants are distinguished as “drunk driving”;
(4) As a conclusion, the accuracy rate of fatigued state is 90.9%, and the drinking state 95.5%. The overall accuracy rate is 94.4%, the precision is 96.92%, and the recall is 95.45%.The risk estimate of resubstitution is 0.057 and the risk estimate of cross-validation is 0.250.

The results show that it is possible to distinguish fatigue from drinking state of the drivers with high accuracy by calculating the SP_SD, LP_AVG, and LP_SD in the curves with 200-m radius and 500-m radius.

**Figure 7:**
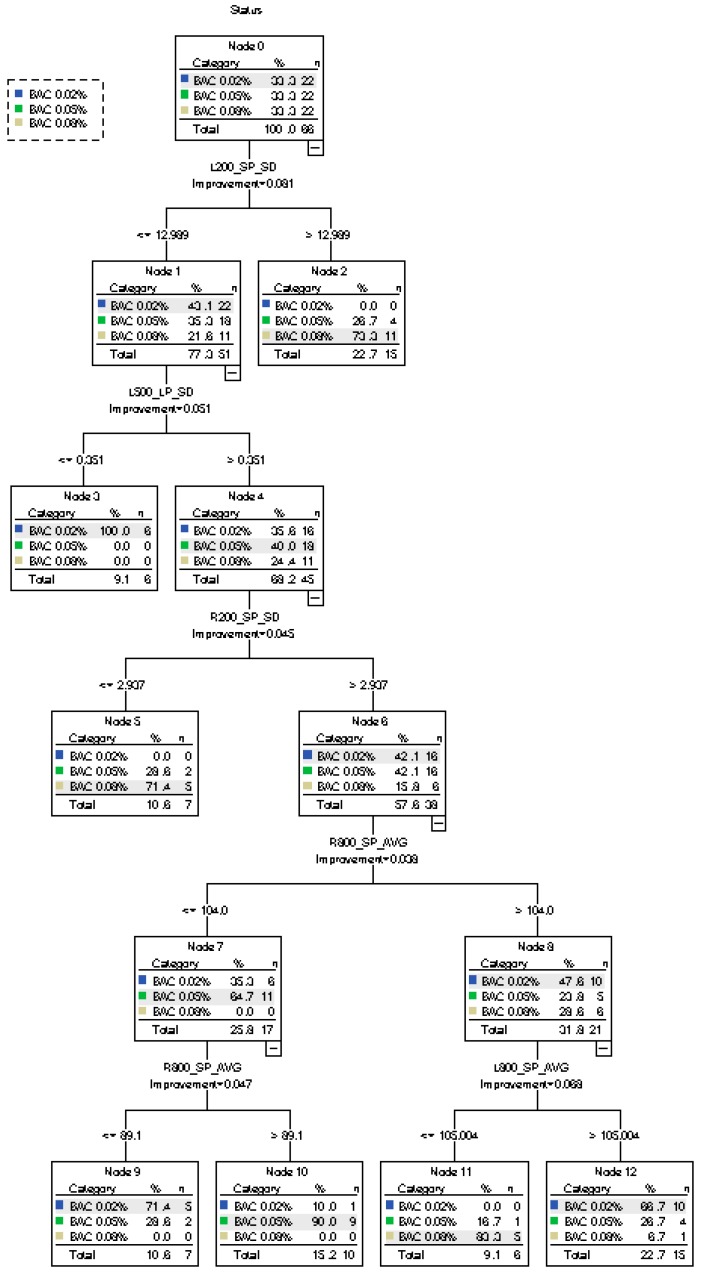
Decision tree diagram for the CART model of various BAC levels.

Figure 7 displays the results of the third CART model of various BAC levels. When the depth of decision tree reached five, the model produced seven terminal nodes. The model’s characteristics are listed as follows:

(1) When L200_SP_SD ≤12.989, 77.3% of the participants are classified to level BAC 0.02%. The rest (22.7%) are classified to level BAC 0.08% since L200_SP_SD >12.989.
(2) When L500_LP_SD ≤0.351, 9.1% of the participants are classified as BAC 0.02%, accurately. Here, 68.2% of the participants are classified as BAC 0.05%. When R200_SP_SD ≤2.937, 10.6% of the participants are classified as BAC 0.08%.
(3) Further classification is performed for BAC 0.02%, BAC 0.05%, and BAC 0.08%, according to R800_SP_AVG and L800_SP_AVG
(4) As a conclusion, the accuracy rates of BAC 0.02% and BAC 0.08% are 95.5%, and the accuracy rate of BAC 0.05% is 40.9%. Since this classification has three categories, the accuracy, precision, and recall of each category are calculated, and the overall evaluation indexes are determined by the mean value of each category. The overall accuracy rate is 77.3%, the precision is 61.32%, and the recall is 80%. The risk estimate of resubstitution is 0.227 and the risk estimate of cross-validation is 0.606.

Compared with the previous two decision trees, the accuracy rate of classifying different drinking levels by driving behaviors is relatively low, but more accurate when classifying the low drinking level (BAC 0.02%) and high drinking level (BAC 0.08%); the misjudgment rate of the medium drinking level (BAC 0.05%) is high. SP_AVG, SP_SD, LP_AVG, and LP_SD in curves with different radii could be used to distinguish different BAC levels.

## 5. Conclusions

In this paper, we built decision trees based on CART algorithm. Different driving states were taken as main factors and driving performance indicators as dependent variables. First, regardless the curvature of different segments, we took the entire route as an entirety, and calculated the mean and standard deviation. The mean and standard deviation of all driving performance indicators are used as independent variables to build the decision trees. Second, driving performance indicators are calculated under straight segments and curves respectively, and are input as independent variables. Then, decision trees with driving performance indicators of six different curves were built respectively. After analyzing all the results, we found that it was necessary to use different driving performance indicators under different road geometries, and the thresholds, therefore, also vary. The accuracy rate of identifying driving states under different road geometries also varies significantly.

Both the road environment and driving performance keep changing during the driving process. Even under the same driving state, changes in road geometries will result in changes in driving performance. We input all the driving performance indicators (28 in total) under seven road geometries (straight, L200, L500, L800, R200, R500, R800) as independent variables to build the decision tree to classify driving states more efficiently. With CART models, it become easier to choose the sensible indicators that can classify driving states under a given road geometry, including L200_LP_SD, S_LP_SD, and L500_ LP_AVG, etc. This research methodology can be used to select indicators efficiently and effectively from massive and multidimensional data bases, and to classify and predict fatigued driving and drunk driving.

At last, to take full advantage of the decision trees in practical application, we reduced the dimension of independent variables and established three decision trees to classify driving states. First, we can classify all the five states into two parts: “abnormal state”, the opposite of the “alert state”. Then, another decision tree was built to classify “fatigued driving” and “drunk driving” in “abnormal states”. The third decision tree is established to classify “drunk driving” with three different BAC levels. Since the decision tree is more efficient and accurate for processing binary data, such a design will improve the efficiency and accuracy of the calculation.

This paper established a three-step decision tree model to identify and classify driving states. It will be a useful reference for future research in this sector. In the coming studies, more methods will be employed to classify indicators and to predict fatigued driving and drunk driving. More driving performance indicators, including steering angle, accelerator position, and brake pressure, will be taken into account. Meanwhile, driver characteristics, including gender, age, driving experience, and individual driving habits will also be taken into consideration as variable factors to increase the accuracy of classification and prediction of risky driving behaviors.

The scope of the future research includes the following directions. Firstly, driving behavior indexes for classification only considered the mean or standard deviation of each road segment, and may cause some information loss. In future studies, In future studies, more time-varying driving behavior indicators would be considered for the identification the fatigue driving and drunk driving in order to improve the accuracy of the classification model. Secondly, the effect of the decision tree is very good when dealing with the data of small or medium-sized samples, but overfitting may occur when the data size increases. At the same time, decision trees can be unstable because small variations in the data might result in a completely different tree being generated. Therefore, in future studies, more ensemble learning algorithms (e.g., the Gradient Boosting Decision Tree, Gradient Boosting Machine) or Random Forest algorithms would be considered to improve the accuracy and applicability of the classification model.

## Figures and Tables

**Table 1:** Results of repeated measures ANOVA in curves for all of driving performance measures [17]. D: curve direction; R: radius; S: drivers’ state.

Performance Measure	Main			Interaction		
	S	D	R	S × D	S × R	S × D × R
SP_AVG	0.000 *	0.019 *	0.000 *	0.828	0.048 *	0.806
LP_AVG	0.000 *	0.000 *	0.248	0.001 *	0.746	0.404
SP_SD	0.000 *	0.224	0.247	0.148	0.001 *	0.004 *
LP_SD	0.000 *	0.089	0.003 *	0.095	0.175	0.183

* *p* < 0.05.

**Table 2:** Correct percentage of predicted classification of the Classification and Regression Tree (CART) model.

Observed	Predicted Percent Correct (%)									
**Roadway** **Geometry**	A	B	C	D	E	F	G	H	I	X
	Overall ^1^	Straight ^2^	Curve ^3^	L200 ^4^	L500	L800	R200	R500	R800	Combined ^5^
Alert	68.2	27.3	86.4	86.4	90.9	77.3	90.9	68.2	54.5	86.4
Fatigue	31.8	68.2	59.1	50.0	36.4	22.7	59.1	81.8	72.7	90.9
BAC 0.02%	86.4	63.6	45.5	90.9	68.2	9.1	68.2	31.8	0.0	50.0
BAC 0.05%	36.4	0.0	81.8	0.0	50.0	77.3	36.4	72.7	68.2	54.5
BAC 0.08%	31.8	77.3	27.3	45.5	18.2	72.7	72.7	45.5	50.0	63.6
**Overall Percentage**	**50.9**	**47.3**	**60.0**	**54.5**	**52.7**	**51.8**	**65.5**	**60.0**	**49.1**	**69.1**

^1^ “Overall” refers to the whole route that does not divide road conditions, including all straight and curve segments. Four independent variables are measured under the whole route: SP_AVG, SP_SD, LP_AVG and LP_SD. ^2^ “Straight” refers to the straight segments, where four independent variables, i.e., S_SP_AVG, S_SP_SD, S_LP_AVG, and S_LP_SD are measured. ^3^ “Curve” refers to the curve segments, where four independent variables, i.e., C_SP_AVG, C_SP_SD, C_LP_AVG, and C_LP_SD, are measured. ^4^ “L200” refers to a curve to the left with a radius of 200 m, where four independent variables, i.e., L200_SP_AVG, L200_SP_SD, L200_LP_AVG, and L200_LP_SD, are measured. ^5^ “Combined” refers to the variables measured under different segments, including SP_AVG, SP_SD, LP_AVG, and LP_SD under the straight segment, and the same indicators under six curve segments, respectively. A total of 4 + 4 × 6 = 28 independent variables are measured. BAC: blood alcohol content.

**Table 3:** Risk estimate of predicted classification of the CART model.

Method	Estimate									
**Roadway** **Geometry**	A	B	C	D	E	F	G	H	I	**X**
	Overall	Straight	Curve	L200	L500	L800	R200	R500	R800	**Combined**
**Resubstitution**	0.491	0.527	0.400	0.455	0.473	0.482	0.345	0.400	0.509	**0.309**
**Cross-Validation**	0.736	0.645	0.700	0.609	0.691	0.755	0.682	0.682	0.709	**0.764**
